# TPVB and general anesthesia affects postoperative functional recovery in elderly patients with thoracoscopic pulmonary resections based on ERAS pathway

**DOI:** 10.1515/tnsci-2022-0305

**Published:** 2023-09-06

**Authors:** Na An, Wenzhe Dong, Guangdong Pang, Yiwei Zhang, Chunling Liu

**Affiliations:** Department of Anesthesia 1, Inner Mongolia People’s Hospital, Hohhot 010020, Inner Mongolia, China; Department of Thyroid Oncology, Inner Mongolia People’s Hospital, Hohhot 010020, Inner Mongolia, China

**Keywords:** enhanced recovery after surgery, thoracic paravertebral block, thoracoscopy, lung cancer, elderly patients, nursing

## Abstract

**Objective:**

Thoracic surgery is easy to cause various perioperative complications, especially in elderly patients, due to their physical weakness and physiological function degeneration. Postoperative cognitive dysfunction is a common complication in elderly patients undergoing thoracic surgery. This study focuses on exploring the effects of thoracic paravertebral block (TPVB) combined with general anesthesia on postoperative functional recovery in elderly patients undergoing thoracoscopic radical resection for lung cancer based on enhanced recovery after surgery (ERAS) pathway.

**Methods:**

A total of 104 patients aged 60 years or older undergoing thoracoscopic radical resection of lung cancer were randomized into the combination group (*n* = 52) and the control group (*n* = 52). Patients in the control group were given general anesthesia alone, while patients in the combination group were given TPVB combined with general anesthesia. All patients applied the ERAS model for the perioperative intervention. Hemodynamic indices (heart rate [HR] and mean arterial pressure [MAP]) before anesthesia (T0), 5 min after thoracoscopic trocar placement (T1), at extubation (T2), 30 min after extubation (T3), and 6 h after the surgery (T4), postoperative analgesia, preoperative and postoperative serum pain stress factors (5-hydroxytryptamine [5-HT], prostaglandin E2 [PGE2], cortisol [Cor], substance P [SP], and norepinephrine [NE]), tumor markers (CYFRA21-1, CEA, and CA50), inflammatory factors (IL-6, TNF-α, and c-reactive protein (CRP)), lung function indicators (forced vital capacity [FVC] and forced expiratory volume in the first second [FEV1]), 6 min walking distance (6MWD), clinical recovery indicators, hospitalization status, and postoperative complications in patients between both groups were compared.

**Results:**

Compared with the control group, patients in the combination group had lower HR and MAP at T1–T4 time points, less intraoperative doses of remifentanil and propofol, less patient-controlled interscalene analgesia compression number 24 h after the surgery, lower visual analogue scale scores 24 h after the surgery, shorter hospitalization time, postoperative off-bed time, postoperative chest tube removal time, postoperative first feeding time and gastrointestinal function recovery time, reduced postoperative serum levels of 5-HT, PGE2, Cor, SP, NE, CYFRA21-1, CEA, CA50, IL-6, TNF-α, and CRP, decreased complications, and higher FVC, FEV1, and 6MWD.

**Conclusion:**

Based on the ERAS pathway, TPVB combined with general anesthesia in thoracoscopic surgery for lung cancer in elderly patients can effectively reduce the patients’ hemodynamic fluctuations, alleviate postoperative pain, accelerate the recovery process, and reduce complications.

## 1 Introduction

Lung cancer is a frequently diagnosed malignancy and a major cause of cancer-relevant mortality around the world with an estimated 1.76 million deaths and 2 million new cases each year [1]. It is classified into various histologic subtypes, such as squamous carcinoma, adenocarcinoma, small cell lung cancer, and non-small cell lung cancer (NSCLC) [2]. Lung cancer, often diagnosed at an advanced stage, possesses a poor prognosis [3]. Moreover, the mean age at diagnosis of lung cancer is rising with increasing age [4]. Even if about half of the patients who are newly diagnosed with NSCLC are at the age of 70 years or older, elder patients are underrepresented in clinical trials [5].

For the therapy of patients with early-stage NSCLC, thoracoscopic resection is an effective choice. It can reduce hospital stay, indwelling time of drainage tube, drainage volume and blood loss, in a high safe manner [6]. Thoracic paravertebral block (TPVB) may be highly beneficial for patients undergoing thoracoscopic lobectomy [7]. It is also reported that TPVB can decrease pain during thoracic surgery [8]. TPVB is an efficient regional anesthetic method that is able to offer significant analgesia to many surgical procedures, including pulmonary surgery [9]. A previous study has demonstrated that TPVB can not only improve postoperative analgesic effects but also reduce serum tumor marker levels in patients who receive thoracoscopic radical resection for lung cancer, and during the process, there are no increasing adverse reactions [10]. In a previous study, it is unearthed that TPVB combined with general anesthesia can alleviate early cognitive function in elderly patients undergoing lobectomy after the surgery. Furthermore, TPVB combined with general anesthesia has better effects [11]. Fast-track surgery, also named enhanced recovery after surgery (ERAS) [12], is effective during the perioperative period of single-hole thoracoscopic radical resection for lung cancer. It can accelerate patients’ recovery, alleviate pain, lower hospital stay length, and decrease hospitalization expenses [13]. In our article, the study focus was on the combined effects of TPVB and general anesthesia on elderly patients undergoing thoracoscopic radical resection of lung cancer under the ERAS model. Consequently, this research was aimed at investigating the effects of TPVB combined with general anesthesia on postoperative functional recovery in elderly patients undergoing thoracoscopic radical resection of lung cancer based on the ERAS pathway.

## 2 Materials and methods

### 2.1 Study subjects

A total of 104 elderly patients with lung cancer who underwent thoracoscopic radical resection of lung cancer in Inner Mongolia People’s Hospital from May 2019 to May 2020 were selected for this study. Patients were randomized into the combination group (*n* = 52) and the control group (*n* = 52). Patients in the control group were given general anesthesia alone, while patients in the combination group were given TPVB combined with general anesthesia. Inclusion criteria: (1) patients’ age ≥60 years, (2) patients clearly diagnosed by preoperative CT scan, enhancement and fiberoptic bronchoscopic biopsy of the chest, and possessed indications for thoracoscopic radical resection of lung cancer, (3) patients received no radiotherapy or chemotherapy before the surgery, and (4) patients without important organ functional disease and liver or kidney system diseases. Exclusion criteria: (1) patients with autoimmune diseases, (2) those unable to cooperate with nursing interventions and observation due to cognitive impairment, and (3) those combined with other malignancies.

### 2.2 Experimental methods

#### 2.2.1 ERAS nursing

##### 2.2.1.1 Preoperative patient health education

Patients received preoperative education and were inquired about medical history and introduced to the ward environment and safety, surgical-related knowledge, surgical risks, and precautions, as well as the concept of rapid rehabilitation surgery, and signed an informed consent form. Patients were advised to eat a diet rich in cellulose, calories, protein, vitamin, and digestible to improve their constitution and enhance surgical tolerance and stress ability. The patients were assessed by the self-rating anxiety scale (SAS) and the self-rating depression scale (SDS) 1 day before the surgery; according to the score of the SAS and SDS, the patients were given corresponding psychological care to eliminate their tension and anxiety. The risk of thrombosis in patients was assessed, for medium to high-risk patients, it was necessary to wear elastic socks and take medication prevention if necessary.

##### 2.2.1.2 Preoperative respiratory function exercise

The patients were taught to perform respiratory function exercises and respiratory function trainer before surgery; 2 mg budesonide and 2 mL of compound ipratropium bromide solution for inhalation were used for respiratory function drugs (3 days before surgery, 3–5 days after surgery to discharge, twice a day).

##### 2.2.1.3 Intraoperative insulation management

(1) Body temperature control: the operating room temperature was controlled at 22–25℃. Hypothermia was actively prevented by warming intravenous fluids or blood transfusions, reducing intraoperative exposure of patients, utilizing thermostatic saline for wound irrigation, reducing heat loss in the surgical field, and closely monitoring patients’ body temperature. (2) Fluid therapy: the goal-directed fluid therapy was employed and the rate and volume of fluid rehydration were strictly controlled, with intraoperative and postoperative fluid rehydration at 1,000–1,500 mL per day.

##### 2.2.1.4 Postoperative analgesia

A multi-modal analgesic regimen was implemented, which was added with prophylactic analgesia on the basis of intravenous self-administered analgesia by intramuscular injection of parecoxib sodium (40 mg) 3 days after the surgery, twice/day for six times.

##### 2.2.1.5 Nutritional support

The patient received a carbohydrate supplement 2 h before surgery and oral feeding started after surgery. Oral nutrition supplement (about 200 mL high-energy food, 2–3 times a day) started from the day of surgery to the day when the patient could feed normally.

##### 2.2.1.6 Postoperative activities

When the patient was conscious and his vital signs were stable, the bed activities could be performed, such as ankle pump exercises as well as upper limb fist clenching, elbow flexion, and chest expansion exercises. On the first postoperative day and before the removal of the chest drainage tube, cycling in bed was performed two times/day for 15 min/time, during which patients should stop immediately if they experienced dyspnea, palpitations, and other discomfort. During the 6–24 postoperative hours, the bedside nurse and the supervising physician assessed the patients’ off-bed activity ability according to their vital signs, consciousness, drainage, and pain degree, and assisted them in early off-bed activities. Early postoperative off-bed activity emphasized the importance of assessment. Therefore, to ensure patient’s safety, the first time out of bed must be done with the assistance of a nurse.

##### 2.2.1.7 Postoperative extubation

After anesthesia and awakening, effective intervention measures such as beating expectoration and medication should be given to the patient. The urinary catheter was removed 6–24 h after the surgery, and when the 24 h drainage was ≤300 mL, the chest drain was removed. Postoperative nursing was provided until discharge.

##### 2.2.1.8 Anesthesia method

In both groups, electrocardiogram, heart rate (HR), blood pressure (BP), and blood oxygen saturation were monitored after the patients entered the surgery room, and central venous pressure and invasive BP were monitored by right internal jugular vein and left radial artery puncture placement under local anesthesia.

Patients in the control group were placed under general anesthesia with tracheal intubation as follows. Anesthesia induction: sequential intravenous injection of 0.06 mg/kg midazolam (Jiangsu Nhwa Pharmaceutical Co., Ltd, State Drug Administration: H10980025), 0.5 μg/kg sufentanil (Yichang Humanwell Pharmaceutical Co., Ltd, State Drug Administration: H20054171), 0.3 mg/kg etomidate (Jiangsu Nhwa Pharmaceutical Co., Ltd, State Drug Administration: H20020511), and 0.8 mg/kg rocuronium bromide (N.V. Organon, State Drug Administration: H20140847). After the patient fell asleep, the oropharynx and vocal cords were sprayed with 5 mL 2% lidocaine (Hubei Jinyao Pharmaceutical Co., Ltd, State Drug Administration: H20133209) under visual laryngoscopy, followed by five hand-controlled breaths and then double-lumen bronchial catheter intubation, and mechanical ventilation was conducted after the fiberoptic bronchoscope was positioned to determine good isolation of both lungs. After changing the position, the catheter position was again confirmed by fibrinoscopy. Volume control mode was utilized for double-lung ventilation: tidal volume 8 mL/kg, *I*:*E* = 1:2, respiratory rate: 12 times/min, keeping end-tidal carbon dioxide (PETCO_2_) at 35–45 mmHg; for single-lung ventilation, peak airway pressure was controlled to not exceed 30 cm H_2_O, respiratory rate: 14–16 times, maintaining PETCO_2_ at 40–50 mmHg. Anesthesia maintenance: target-controlled infusion of propofol (Guangdong Jiabo Pharmaceutical Co., Ltd, State Drug Administration: H20051842) and remifentanil (Yichang Humanwell Pharmaceutical Co., Ltd, State Drug Administration: H20030197) was implemented. The target plasma concentrations were 2–4 and 2–4 ng/mL, respectively. The amplitude of BP fluctuations was maintained at no more than 20% of the basal value, and rocuronium bromide (N.V. Organon, State Drug Administration: H20140847) was intermittently administered intravenously to maintain muscle relaxation, and intraoperative bispectral index values of 40–60 were maintained. When the chest cavity was closed, the anesthesia was stopped and 50 mg flurbiprofen ester (Wuhan Docan Pharmaceutical Co., Ltd, State Drug Administration: H20183054) was injected, and a patient-controlled analgesia (PCA) was connected, formulated with 2 μg/kg sufentanil + 16 mg to 100 mL ondansetron (QILU Pharmaceutical Co., Ltd, State Drug Administration: H10970065), with a loading dose of 2 mL, a background dose of 2 mL/h, a PCA of 2 mL, and a lock time of 15 min. No routine antagonism of inotropic drugs was utilized and the patients were sent to the anesthesia recovery room after recovery of spontaneous breathing.

Patients in the combination group were given TPVB combined with general anesthesia of tracheal intubation, and the details are as follows. The patients were placed in a lateral position before induction of anesthesia, with the operative side upward, and the paravertebral space of T4, T7, and T9 was selected for a three-point block according to the surgical site. The T4 spinous process was palpated and marked, and the probe was put at the position of the T4 spinous process perpendicular to the posterior midline, showing the T4 spinous process and the T5 transverse process, and then the probe was moved laterally, which showed that the space enclosed by the transverse process, the suprascapular ligament and the pleura was the paravertebral space. An in-plane technique was employed to enter the needle. When the tip of the needle reached the paravertebral space of T4, the local anesthetic (10 mL 0.5% ropivacaine [Astra Zeneca AB, State Drug Administration: H20140763]) was injected slowly after backdrawing without blood and gas. The mural pleura was seen to press down ventrally during the injection, and the local anesthetic spread in the paravertebral space, and 10 mL 0.5% ropivacaine was injected in the same way at T7 and T9, and after 20 min the induction was performed when the level of anesthesia reached from T3 to T10. Anesthesia induction and maintenance were the same as those in the control group.

##### 2.2.1.9 Pulmonary function assessment

Pulmonary function was assessed by implementing a portable spirometer (Contec SP10, Shanghai, China), and each test was performed three times, and the highest value was taken for analysis. The observation indicators were forced vital capacity (FVC) and forced expiratory volume in the first second (FEV1).

##### 2.2.1.10 6 min walking distance (6MWD)

A 30 m long ward corridor was selected as the assessment site, with the starting and return points marked and the necessary test equipment prepared. Patients avoided strenuous exercise for 2 h before the test, and the distance walked in 6 min was recorded at the end of the test. 6MWD was scheduled after the pulmonary function assessment.

### 2.3 Observation indicators

All patients and outcome assessors were blinded to the intervention. (1) Hemodynamic indicators: HR and mean arterial pressure (MAP) were recorded before anesthesia (T0), 5 min after thoracoscopic trocar placement (T1), at extubation (T2), 30 min after extubation (T3), and 6 h after the surgery (T4). (2) Analgesia: the number of patient-controlled interscalene analgesia (PCIA) analgesic pump presses 24 h after the surgery and the dosage of intraoperative analgesic drugs propofol and remifentanil in both groups were recorded. (3) Pain intensity: visual analogue scale (VAS) scores were utilized to evaluate the degree of pain at 6, 12, and 24 h after the surgery in both groups. The scores ranged from 0 to 10, with higher scores demonstrating severe pain. (4) Clinical recovery indicators and hospitalization: hospitalization time, postoperative off-bed time, postoperative chest tube removal time, postoperative first feeding time, and gastrointestinal function recovery time (bowel sound recovery time) of patients in both groups were statistically recorded. (5) Pain stress factors: the levels of pain mediators (prostaglandin E2 [PGE2] and 5-hydroxytryptamine [5-HT]) were measured by fluorescence spectrophotometry, and stress indicators (cortisol [Cor], norepinephrine [NE], and substance P [SP]) were tested by radioimmunoassay. (6) Tumor markers: the contents of tumor markers (CYFRA21-1, CEA, and CA50) in both groups were assessed utilizing electrochemiluminescence immunoassay 1 day before and 7 days after the surgery. (7) Inflammatory factors: the levels of IL-6, TNF-α, and c-reactive protein (CRP) were measured 1 day before the surgery and 7 days after the surgery utilizing enzyme-linked immunosorbent assay. (8) Pulmonary function indicators and 6MWD: pulmonary function instrument was utilized to measure FEV1, FVC, and record 6MWD 1 day before and 7 days after the surgery. (9) Postoperative complications: bleeding, infection, pulmonary atelectasis, and poor incision healing.

### 2.4 Statistics

SPSS 22.0 statistical software was employed for data processing. Measurement data were depicted as mean ± standard deviation and analyzed using independent samples *t*-test or repeated measures analysis of variance (ANOVA). Enumeration data were expressed as number of cases or percentage, and *χ* ^2^ test was implemented for comparisons between groups. *P* < 0.05 indicated a statistically significant difference.


**Ethical approval:** The research related to human use has been complied with all the relevant national regulations, institutional policies and in accordance with the tenets of the Helsinki Declaration, and has been approved by the authors’ institutional review board or equivalent committee. This research was under the approval of the Ethics Committee of Inner Mongolia People’s Hospital (approval number: 20190213).
**Informed consent:** Informed consent has been obtained from all individuals included in this study.

## 3 Results

### 3.1 General data

The general information of patients in the control and combination groups was compared. The average age of patients in the control group was 67.48 ± 5.61 years, and there were 39 males and 13 females, while the average age of patients in the combination group was 67.31 ± 5.77 years, and there were 35 males and 17 females. No significant differences were presented in age, gender, body mass index, location of operated lung lobes, smoking status, clinical stage, and pathological classification between the two groups of lung cancer patients (*P* > 0.05; Table 1), which were comparable.

**Table 1:** General data between the two groups of patients

	The control group (*n* = 52)	The combination group (*n* = 52)	*P* value
Age (years)	67.48 ± 5.61	67.31 ± 5.77	0.879
Gender			0.517
Male	39	35	
Female	13	17	
Body mass index (kg/m^2^)	26.19 ± 2.63	26.45 ± 2.27	0.591
Location of operated lung lobes			0.579
Upper lobe	32	28	
Middle lobe	5	4	
Lower lobe	15	20	
Smoking status			0.787
Never smoking	41	38	
Occasionally smoking	3	4	
Always smoking	8	10	
Clinical stage			0.587
Stage Ⅰ	20	20	
Stage Ⅱ	29	31	
Stage Ⅲ	3	1	
Pathological classification			0.980
Squamous cell carcinoma	22	21	
Adenocarcinoma	28	29	
Others	2	2	

### 3.2 Perioperative hemodynamic indicator levels

By comparing the levels of hemodynamic indicators HR and MAP between the control and the combination groups, HR and MAP were analyzed by repeated measures ANOVA, and the differences between the two groups at different time points were significant (HR: *F* = 2401.37, *P* < 0.001; MAP: *F* = 1537.23, *P* < 0.001), with an interaction between the interventions and the time points (HR: *F* = 74.64, *P* < 0.001; MAP: *F* = 56.66, *P* < 0.001). An in-depth comparison showed that the HR at T1 time point was lower than the T0 time point and the HR at T2–T4 time points was higher than the T0 time point both in the control and observation groups; the MAP at T1–T4 time points was higher than the T0 time point both in the control and observation groups (*P* < 0.05). HR and MAP at T1–T4 time points of the combination group were reduced in contrast with those of the control group (*P* < 0.05, Table 2). It is suggested that TPVB combined with general anesthesia in thoracoscopic surgery for lung cancer in elderly patients can maintain the hemodynamic stability of the patients based on the ERAS pathway.

**Table 2:** Perioperative hemodynamic indicator levels between the two groups of patients

Indicators	Time	The control group (*n* = 52)	The combination group (*n* = 52)	*P* value
HR (time/min)	T0	75.78 ± 7.26	75.11 ± 7.35	0.641
	T1	70.12 ± 5.62*	65.36 ± 4.66*	<0.001
	T2	83.69 ± 4.03*	78.56 ± 5.52*	<0.001
	T3	84.40 ± 5.38*	79.37 ± 6.21*	<0.001
	T4	80.06 ± 6.03*	77.96 ± 4.27*	0.043
MAP (mmHg)	T0	90.79 ± 10.24	88.17 ± 10.52	0.201
	T1	105.87 ± 7.63*	96.07 ± 9.07*	<0.001
	T2	110.53 ± 5.59*	104.12 ± 5.18*	<0.001
	T3	108.52 ± 6.28*	103.37 ± 6.34*	<0.001
	T4	115.71 ± 5.65*	105.61 ± 5.62*	<0.001

Note: * *P* < 0.05 vs T0 within the group.

### 3.3 Intraoperative and postoperative anesthesia

The comparison of intraoperative and postoperative anesthesia in patients between the control and the combination groups revealed that the intraoperative doses of remifentanil and propofol in the combination group were less versus those in the control group (*P* < 0.05), and after 24 h of the surgery, PCIA compression number in the combination group was less versus that in the control group (*P* < 0.05, Table 3).

**Table 3:** Intraoperative and postoperative anesthesia between the two groups of patients

	Intraoperative remifentanil dose (mg)	Intraoperative propofol dose (mg)	Postoperative analgesic pump press number (times)
The control group (*n* = 52)	2.61 ± 0.55	741.08 ± 50.79	10.98 ± 2.88
The combination group (*n* = 52)	1.98 ± 0.28	624.41 ± 43.66	3.24 ± 1.46
*P* value	<0.001	<0.001	<0.001

### 3.4 Postoperative VAS scores

The postoperative VAS scores in patients were compared between the control and the combination groups. VAS scores were analyzed by repeated measures ANOVA, and the differences between the two groups at different time points were significant (*F* = 4187.41, *P* < 0.001), with an interaction between the interventions and the time points (*F* = 61.61, *P* < 0.001). An in-depth comparison demonstrated that the VAS scores of patients in the combination group at 6, 12 and 24 h after the surgery were lower versus those of patients in the control group (*P* < 0.05, Table 4). It is summarized that TPVB combined with general anesthesia in thoracoscopic surgery for lung cancer in elderly patients can relieve postoperative pain of the patients based on the ERAS pathway.

**Table 4:** Postoperative VAS scores between the two groups of patients

	6 h after the surgery	12 h after the surgery	24 h after the surgery
The control group (*n* = 52)	3.74 ± 0.92	2.61 ± 1.05	2.08 ± 0.79
The combination group (*n* = 52)	2.77 ± 0.88	1.98 ± 0.78	1.41 ± 0.66
*P* value	<0.001	<0.001	<0.001

### 3.5 Clinical recovery indicators and hospitalization

The comparison of clinical recovery indicators and hospitalization in patients between the control and the combination groups disclosed that the patients in the combination group possessed shorter hospital stay, postoperative off-bed time, postoperative chest tube removal time, postoperative first feeding time, and gastrointestinal function recovery time compared with those in the control group (*P* < 0.05, Table 5).

**Table 5:** Clinical recovery indicators and hospitalization between the two groups of patients

	Hospital stay (days)	Postoperative off-bed time (h)	Postoperative chest tube removal time (days)	Postoperative first feeding time (h)	Gastrointestinal function recovery time (h)
The control group (*n* = 52)	8.56 ± 0.84	42.16 ± 5.17	5.46 ± 0.73	12.57 ± 1.78	78.43 ± 6.28
The combination group (*n* = 52)	5.07 ± 0.63	21.31 ± 1.33	3.12 ± 0.61	6.23 ± 1.09	71.53 ± 5.34
*P* value	<0.001	<0.001	<0.001	<0.001	<0.001

### 3.6 Serum pain stress indicators

The levels of preoperative and postoperative serum pain stress factors (5-HT, PGE2, Cor, SP, and NE) in the control and combination groups were compared. 5-HT, PGE2, Cor, SP, and NE levels were analyzed by repeated measures ANOVA, and the differences were significant between the two groups before and after surgery (5-HT: *F* = 4862.46, *P* < 0.001; PGE2: *F* = 346579.43, *P* < 0.001; Cor: *F* = 8623.22, *P* < 0.001; SP: *F* = 2290.61, *P* < 0.001; NE: *F* = 12716.13, *P* < 0.001); there was an interaction between group and time point (5-HT: *F* = 624.38, *P* < 0.001; PGE2: *F* = 3236.35, *P* < 0.001; Cor: *F* = 500.62, *P* < 0.001; SP: *F* = 726.24, *P* < 0.001; NE: *F* = 2413.62, *P* < 0.001). An in-depth comparison revealed that after the surgery, the levels of 5-HT, PGE2, Cor, SP, and NE in the serum of patients in the two groups were reduced, and 5-HT, PGE2, Cor, SP, and NE levels in the combination group were lower versus those in the control group (*P* < 0.05, Table 6). It is summarized that TPVB combined with general anesthesia in thoracoscopic surgery for lung cancer in elderly patients can reduce the serum levels of pain stress factors of the patients based on the ERAS pathway.

**Table 6:** Serum pain stress indicators between the two groups of patients before and after the surgery

Indicators	Time	The control group (*n* = 52)	The combination group (*n* = 52)	*P* value
5-HT (μmol/mL)	Before the surgery	0.41 ± 0.06	0.42 ± 0.06	0.397
	After the surgery	0.94 ± 0.14*	0.67 ± 0.06*	<0.001
PGE2 (pg/mL)	Before the surgery	106.01 ± 11.45	105.91 ± 10.55	0.963
	After the surgery	198.61 ± 9.39*	182.19 ± 10.37*	<0.001
Cor (ng/mL)	Before the surgery	67.14 ± 13.12	67.40 ± 12.33	0.917
	After the surgery	113.75 ± 8.61*	95.91 ± 8.63*	<0.001
SP (μg/mL)	Before the surgery	3.44 ± 0.58	3.49 ± 0.53	0.647
	After the surgery	17.57 ± 2.88*	7.44 ± 1.99*	<0.001
NE (pg/mL)	Before the surgery	1.55 ± 0.28	1.57 ± 0.36	0.753
	After the surgery	4.78 ± 0.55*	2.84 ± 0.26*	<0.001

Note: * *P* < 0.05 vs before the surgery.

### 3.7 Serum tumor marker levels

The levels of preoperative and postoperative serum tumor marker factors (CYFRA21-1, CEA, and CA50) were compared in the control and combination groups. CYFRA21-1, CEA, and CA50 levels were analyzed by repeated measures ANOVA, and the differences were significant between the two groups before and after surgery (CYFRA21-1: *F* = 228723.26, *P* < 0.001; CEA: *F* = 1742.02, *P* < 0.001; CA50: *F* = 64912.36, *P* < 0.001); there was an interaction between group and time point (CYFRA21-1: *F* = 676.68, *P* < 0.001; CEA: *F* = 8.54, *P* < 0.001; CA50: *F* = 291.77, *P* < 0.001). An in-depth comparison revealed that after the surgery, the levels of CYFRA21-1, CEA, and CA50 in both groups were decreased, and CYFRA21-1, CEA, and CA50 levels in the combination group were lower versus those in the control group (*P* < 0.05, Table 7). It is concluded that TPVB combined with general anesthesia in thoracoscopic surgery for lung cancer in elderly patients can reduce the serum levels of tumor marker factors of the patients based on the ERAS pathway.

**Table 7:** Serum tumor marker levels between the two groups of patients before and after the surgery

Indicators	Time	The control group (*n* = 52)	The combination group (*n* = 52)	*P* value
CYFRA21-1 (ng/mL)	Before the surgery	5.41 ± 0.66	5.47 ± 0.47	0.595
	After the surgery	2.54 ± 0.64*	2.27 ± 0.56*	0.024
CEA (ng/mL)	Before the surgery	26.01 ± 6.05	25.91 ± 6.55	0.936
	After the surgery	10.61 ± 2.49*	8.19 ± 2.07*	<0.001
CA50 (U/mL)	Before the surgery	27.14 ± 4.12	27.40 ± 4.33	0.754
	After the surgery	12.75 ± 3.60*	10.94 ± 3.63*	0.012

Note: * *P* < 0.05 vs before the surgery.

### 3.8 Serum inflammatory factor contents

The levels of preoperative and postoperative serum inflammatory factor contents (IL-6, TNF-α, and CRP) in the control and combination groups were compared. IL-6, TNF-α, and CRP levels were analyzed by repeated measures ANOVA, and the differences were significant between the two groups before and after surgery (IL-6: *F* = 4530.20, *P* < 0.001; TNF-α: *F* = 73423.27, *P* < 0.001; CRP: *F* = 580.48, *P* < 0.001); there was an interaction between group and time point (IL-6: *F* = 121.96, *P* < 0.001; TNF-α: *F* = 2461.95, *P* < 0.001; CRP: *F* = 49.04, *P* < 0.001). An in-depth comparison suggested that after the surgery, serum IL-6, TNF-α, and CRP contents in the two groups were reduced, and serum IL-6, TNF-α, and CRP contents in the combination group were lower versus those in the control group (*P* < 0.05, Table 8). It is demonstrated that TPVB combined with general anesthesia in thoracoscopic surgery for lung cancer in elderly patients can reduce the serum levels of inflammatory factors of the patients based on ERAS pathway.

**Table 8:** Serum inflammatory factor contents between the two groups of patients before and after the surgery

Indicators	Time	The control group (*n* = 52)	The combination group (*n* = 52)	*P* value
IL-6 (ng/mL)	Before the surgery	75.64 ± 3.23	76.49 ± 3.82	0.223
	After the surgery	177.34 ± 17.69*	149.52 ± 15.71*	<0.001
TNF-α (pg/m)	Before the surgery	0.81 ± 0.34	0.79 ± 0.36	0.771
	After the surgery	2.23 ± 0.36*	1.77 ± 0.42*	<0.001
CRP (mg/L)	Before the surgery	8.22 ± 1.14	8.27 ± 1.64	0.857
	After the surgery	34.82 ± 12.04*	22.89 ± 7.42*	<0.001

Note: * *P* < 0.05 vs before the surgery.

### 3.9 Pulmonary function indicators and 6MWD

The indicators of pulmonary function (FVC and FEV1) and 6MWD before and after surgery in the control and combination groups were compared. FVC, FEV1, and 6MWD were analyzed by repeated measures ANOVA, and the differences were significant between the two groups before and after surgery (FVC: *F* = 4410.67, *P* < 0.001; FEV1: *F* = 809.19, *P* < 0.001; 6MWD: *F* = 84132.86, *P* < 0.001); there was an interaction between group and time point (FVC: *F* = 76.73, *P* < 0.001; FEV1: *F* = 9.31, *P* < 0.001; 6MWD: *F* = 277.42, *P* < 0.001). An in-depth comparison suggested that after the surgery, FVC, FEV1, and 6MWD were reduced in patients in the two groups; FVC, FEV1, and 6MWD in the combination group were higher in contrast with those in the control group (*P* < 0.05, Table 9). It is implied that TPVB combined with general anesthesia in thoracoscopic surgery for lung cancer in elderly patients can improve the pulmonary function of the patients after the surgery based on the ERAS pathway.

**Table 9:** Pulmonary function indicators and 6MWD between the two groups of patients before and after the surgery

Indicators	Time	The control group (*n* = 52)	The combination group (*n* = 52)	*P* value
FEV1 (L)	Before the surgery	2.49 ± 0.57	2.45 ± 0.68	0.746
	After the surgery	1.16 ± 0.44*	1.43 ± 0.46*	0.003
FVC (L)	Before the surgery	2.24 ± 0.73	2.25 ± 0.72	0.944
	After the surgery	1.05 ± 0.36*	1.29 ± 0.32*	<0.001
6MWD (m)	Before the surgery	517.39 ± 15.27	521.33 ± 15.77	0.199
	After the surgery	416.36 ± 13.84*	431.27 ± 20.30*	<0.001

Note: * *P* < 0.05 vs before the surgery.

### 3.10 Postoperative complications

The comparison of postoperative complications of patients in the control and combination groups disclosed that the overall postoperative complication rate of the combination group was 5.77%, which was lower versus that of the control group at 23.08% (*P* < 0.05, Table 10). It is demonstrated that TPVB combined with general anesthesia in thoracoscopic surgery for lung cancer in elderly patients has few postoperative adverse effects based on the ERAS pathway.

**Table 10:** Postoperative complications between the two groups of patients (*n* [%])

Group	Bleeding	Lung infection	Pulmonary atelectasis	Poor incision healing	Continuous air leakage	Pleural effusion	Total	*P* value
The control group (*n* = 52)	3 (5.77)	2 (3.85)	3 (5.77)	2 (3.85)	1 (1.92)	1 (1.92)	12 (23.08)	0.023
The combination group (*n* = 52)	1 (1.92)	1 (1.92)	1 (1.92)	0	0	0	3 (5.77)	

## 4 Discussion

Lung cancer is one of the leading causes of cancer-relevant incidence and mortality around the world [14]. As previously described, ERAS is safe and useful in lung cancer thoracoscopic radical resection, which accelerates recovery and diminishes complications [13]. This study focused on the effects of TPVB combined with general anesthesia on postoperative functional recovery in elderly patients undergoing thoracoscopic radical resection for lung cancer based on the ERAS pathway.

As previously reported, TPVB functions as a protective prognostic factor for patients undergoing lung cancer resection. It can not only prolong survival time but also promote the prognosis of patients who undergo surgical resection for lung cancer [15]. In a previous study, it is shown that for patients treated with TPVB, MAP and HR at 5, 10, and 30 min after extubation are decreased [16], which is consistent with our findings. In our article, we found that HR and MAP at T1–T4 time points of the combination group were reduced in contrast with those of the control group. Increasing evidence has suggested that TPVB combined with general anesthesia exhibits excellent analgesic effects in contrast with general anesthesia alone [7]. TPVB can lower the rate of required analgesia in chest tube duration and the frequency of PCA pressing in 24 h [8]. TPVB, but not general anesthesia, decrease the intraoperative dose of remifentanil, which indicates potential synergistic effects of TPVB to affect patients’ survival [15]. In the meantime, TPVB has reduced the dosage of sufentanil during the operation, the later time of first pressing the PCA pump after the operation and a smaller number of pressing the PCA pump within 48 h after the operation [10]. In our study, it was found that the intraoperative doses of remifentanil and propofol in the combination group were less, and after 24 h of the surgery, the PCIA compression number in the combination group was also less versus those in the control group. In addition, VAS scores of patients in the combination group at 6, 12 and 24 h after the surgery were reduced versus those of patients in the control group. These findings were also observed in previous studies. For instance, it is reported that TPVB could reduce pain undergoing thoracic surgery [8]. Another study has revealed that in the TPVB group, postoperative VAS scores at rest and coughing decrease 6 and 12 h [10]. In addition, patients in the TPVB group possess lower VAS at rest or coughing [16].

Subsequently, we measured clinical recovery indicators, hospitalization, serum pain stress indicators, and serum tumor marker levels and found that the patients in the combination group had shorter hospital stay, postoperative off-bed time, postoperative chest tube removal time, postoperative first feeding time, gastrointestinal function recovery time, reduced 5-HT, PGE2, Cor, SP, and NE levels, and lower CYFRA21-1, CEA, and CA50 levels. It is also reported that the TPVB group possesses lower serum tumor marker levels 24 h postoperatively [10]. Moreover, previous research has demonstrated that TPVB could promote postoperative analgesia, repressing perioperative stress and inflammation [11]. In our article, we found that in the combination group, serum IL-6, TNF-α, and CRP contents were lower, and FVC, FEV1, and 6MWD levels were higher. As for postoperative complications, the overall postoperative complication rate of the combination group was decreased than that of the control group. In a study conducted by Tong et al., general anesthesia combined with TPVB can decrease the incidence of postoperative pulmonary complications, mostly reducing postoperative atelectasis, by reducing postoperative pain in geriatric patients undergoing thoracic surgery compared with general anesthesia alone [8].

Although previous articles have demonstrated the use of TPVB alone or in combination with general anesthesia in postoperative functional recovery in elderly patients undergoing thoracoscopic radial recovery for lung cancer, this study was even more specific about this content, including hemodynamic indices (HR and MAP) before anesthesia (T0), 5 min after thoracoscopic trocar placement (T1), at extubation (T2), 30 min after extubation (T3), and 6 h after the surgery (T4), postoperative analgesia, preoperative and postoperative pain stress factors (5-HT, PGE2, Cor, SP, and NE), preoperative and postoperative tumor markers (CYFRA21-1, CEA, and CA50), preoperative and postoperative inflammatory factors (IL-6, TNF-α, and CRP), preoperative and postoperative lung function indicators (FVC and FEV1), 6MWD, clinical recovery indicators, hospitalization status, and postoperative complications in TPVB combined with general anesthesia in thoracoscopic surgery for lung cancer in elderly patients. In the meantime, all these aspects were conducted on the basis of the ERAS model, which was the novelty of our article.

In summary, this research demonstrates that TPVB combined with general anesthesia in thoracoscopic surgery for lung cancer in elderly patients can effectively lower the patients’ hemodynamic fluctuations, alleviate postoperative pain, accelerate recovery processes, reduce serum inflammatory factor levels and tumor marker levels, improve analgesic effects, and promote rapid recovery on the basis of the ERAS model. This study lays a foundation to study the impact of TPVB combined with general anesthesia in elderly patients undergoing thoracoscopic surgery for lung cancer. However, further exploration is necessary to further convince our findings. In addition, long-term follow-up of the patients should be performed in future studies to know the 5-year survival rates of two groups of patients.
